# Disease Resistance in the Drywood Termite, *Incisitermes schwarzi*: Does Nesting Ecology Affect Immunocompetence?

**DOI:** 10.1673/031.010.4401

**Published:** 2010-05-08

**Authors:** Daniel V. Calleri, Rebeca B. Rosengaus, James F.A. Traniello

**Affiliations:** ^1^ Department of Biology, Boston University, 5 Cummington Street, Boston, MA 02215-2406, USA; ^2^ Department of Biology, Northeastern University, 134 Mugar Life Science Building, 360 Huntington Avenue, Boston, MA 021 15-5000

**Keywords:** ecological immunology, entomopathogenic fungus, herd immunity, infection control, microbial load

## Abstract

Termites live in nests that can differ in microbial load and thus vary in degree of disease risk. It was hypothesized that termite investment in immune response would differ in species living in nest environments that vary in the richness and abundance of microbes. Using the drywood termite, *Incisitermes schwarzi* Banks (Isoptera: Kalotermitidae), as a model for species having low nest and cuticular microbial loads, the susceptibility of individuals and groups to conidia of the entomopathogenic fungus, *Metarhizium anisopliae* Sorokin (Hypocreales: Clavicipitaceae), was examined. The survivorship of *I. schwarzi* was compared to that of the dampwood termite, *Zootermopsis angusticollis* Hagen (Termopsidae), a species with comparatively high microbial loads. The results indicated that *I. schwarzi* derives similar benefits from group living as *Z. angusticollis:* isolated termites had 5.5 times the hazard ratio of death relative to termites nesting in groups of 25 while termites in groups of 10 did not differ significantly from the groups of 25. The results also indicated, after controlling for the influence of group size and conidia exposure on survivorship, that *Z. angusticollis* was significantly more susceptible to fungal infection than *I. schwarzi*, the former having 1.6 times the hazard ratio of death relative to drywood termites. Thus, disease susceptibility and individual investment in immunocompetence may not be dependent on interspecific variation in microbial pressures. The data validate prior studies indicating that sociality has benefits in infection control and suggest that social mechanisms of disease resistance, rather than individual physiological and immunological adaptations, may have been the principle target of selection related to variation in infection risk from microbes in the nest environment of different termite species.

## Introduction

Social insects provide a diverse array of model systems to examine the ecological immunology and sociobiology of disease resistance (Pie et al. 2005; Schmid-Hempel 2005; Cremer et al. 2007; Ugelvig and Cremer 2007). The study of comparative immunity is particularly important to understand the evolution of disease resistance because the induction and maintenance of immunity are costly (Rolff and Siva-Jothy 2003; SchmidHempel 2003) and because immune function is considered to be an adaptive life-history trait (Schmid-Hempel 2005). Investment in immunity should therefore be dependent on the risk of contracting disease: species with reduced pathogen pressure should show reduced investment in immunocompetence. However, the role of interspecific variability in pathogen pressure as a selective agent for adaptive variation in disease resistance has received little attention.

In termites, immune defense is a particularly important life-history trait. Termite social evolution is associated with life type (Abe 1987); the nesting and feeding biology of soiland decayed wood-dwelling species may encourage the proliferation of pathogens relative to that of drywood species. For all termite life types, nestmate density and frequent social interactions among colony members could increase the probability of disease transmission. Termite nests are inhabited by a diverse array of microbes (Hendee 1933, 1934; Meiklejhon 1965; Sands 1969; Keya et al. 1982; Cruse 1988). The drywood termite, *Incisitermes schwarzi* Banks (Isoptera: Kalotermitidae), and the dampwood termite, *Zootermopsis angusticollis* Hagen (Termopsidae), are one-piece nesters that colonize dead wood and are similar in colony size and life history (Castle 1934; Luykx 1986). However, these species have substantial differences in their nesting ecology that could impact exposure to parasites and pathogens: *I. schwarzi* is found most often in dry, dead, intact branches (Collins 1969; Abe 1987; Eggleton 2000), whereas *Z. angusticollis* generally colonize decayed moist wood in contact with leaf litter and/or soil (Castle 1934; Collins 1969; Eggleton 2000). In addition, *Incisitermes* is more tolerant of desiccation than *Zootermopsis* and requires less moisture (Collins 1969), which likely affects microbe development. The dry wood exploited by *I. schwarzi* does not appear to favor the growth of bacteria and fungi (Hendee 1933, 1934; Ignoffo 1992). In fact, Rosengaus et al. (2003) found that *I. schwarzi* has significantly lower nest and cuticular loads of culturable microbial strains than *Z. angusticollis* (average nest load = 58 vs. 824 colony forming units; average cuticular load 4 v. 190 colony forming units). Contact with soil microbes and the habit of nesting in moist, decayed wood may thus have influenced the diversity and abundance of nest microbes and the nature of pathogen challenges. The question as to whether such differences in the nest environment selected for variation in individual and social mechanisms of disease resistance in these two termite species, however, remains unanswered.

Is disease susceptibility in *I. schwarzi* and *Z. angusticollis* associated with variation in microbial loads present in their nests? Is disease susceptibility in the drywood termite *I. schwarzi* decreased by group living as in the dampwood *Z. angusticollis*? Here, the survival of isolated and grouped *I. schwarzi* following low- and high-dose exposures of fungal conidia was examined to estimate immune function, assess disease susceptibility, infer investment in immunocompetence, and determine the role of sociality in infection control in a drywood termite. By using body mass-corrected doses of conidia the results were compared with resistance in *Z. angusticollis* to determine if differences in survivorship following pathogen exposure correlated with variation in nest microbial load.

## Materials and Methods

### Collection and maintenance of termites

Colonies of the drywood termite *I. schwarzi* (*n* = 4, approximately 100–250 individuals) were collected on Grass Key and Key West, Florida in March 2003. Wood containing termites was placed in open Fluon®-lined plastic boxes (50 × 30 × 20 cm). Stock colonies were reared in the laboratory at 25° C and lightly sprayed with water once a month. Termites were removed from their colonies and used for experiments during August and September 2003.

Colonies of *Z. angusticollis* (*n* = 19, approximately 500–1000 individuals) were collected from Redwood East Bay Regional Park, Oakland, California and the Pebble Beach Resort, Monterey, California during July 1999. Log nests containing termites were sectioned and transferred to plastic tubs (50 × 30 × 20 cm) lined with moist paper towels. Decayed wood was added periodically as a supplementary food source. Stock colonies were reared in the laboratory at 25° C and sprayed liberally with water once a week to ensure a high level of moisture. Termites were removed from their colonies and used for experiments during September and October 1999.

### Preparation of conidia suspensions

The entomopathogenic fungus *Metarhizium anisopliae* Sorokin (Hypocreales: Clavicipitaceae) (original source: American Type Culture Collection, batch 93–09, media 325, ATCC #90448) was used as a model pathogen. *M. anisopliae* is an entomopathogenic fungus (Tanada and Kaya 1993) that naturally occurs with a number of soil-dwelling termites (Zoberi 1995; Milner et al. 1998) and can induce mortality in drywood species (Nasr and Moein 1997; Siderhurst et al. 2005). A stock Tween 80 conidia suspension containing 6.4 × 10^8^ conidia/ml was freshly prepared according to Rosengaus et al. (1998). The average germination rate (± S.D.) of conidia was 97.4 ± 6.0% (*n* = 30 fields of vision).

### Determination of body mass-corrected dosage

To compare the susceptibility of *I. schwarzi* and *Z. angusticollis*, conidia dosage was corrected for body mass according to the following protocol. *Z. angusticollis* nymphs (*n* = 10) were allowed to walk freely for 1 h as a group inside a Petri dish (100 × 15 mm) lined with filter paper (Whatman Qualitative no. 5, particle retention > 2.5 µm) moistened with 1.0 ml of a suspension containing 2 × 10^8^ conidia/ml (high dose) or 6 × 10^6^ conidia/ml (low dose) (Rosengaus et al. 1998). Immediately after exposure, each termite was placed in a 1.0 ml microcentrifuge tube with 1.0 ml of Tween 80 solution, vortexed, and then centrifuged at 300 × g at 4° C for 20 min. Next, the termite was removed, the pellet redistributed using the vortex, and a sample of the wash was placed on a hemocytometer to determine the number of conidia washed from the cuticle of each individual sampled. The average mass of *Z. angusticollis* was approximately three times that of *I. schwarzi* (average ± S.D. = 0.045 ± 0.012 g, *n* = 25 nymphs and 0.014 ± 0.005 g, respectively; n = 25 instars 6, 7 and nymph). The resulting average conidia load recorded after washes for *Z. angusticollis* exposed to a high (1.5 × 10^5^ ± 6.7 × 10^4^, *n* = 10 termites) or low dose (9.2 × 10^4^ ± 1.9 × 10^4^, *n* = 10 termites) of conidia were divided by three to arrive at the appropriate conidia loads for *I. schwarzi.*

To determine exposure concentrations that would produce the desired conidia loads, *I. schwarzi* were allowed to walk freely for 1 h in groups of 10 composed of mixed developmental stages (instars 6, 7 and nymphs) in a Petri dish (60 × 15 mm) lined with filter paper (Whatman Qualitative no. 5, particle retention > 2.5 µm) moistened with 0.5 ml of a 6.4 × 10^8^, 6.4 × 10^7^, 5.8 × 10^6^, 6.2 × 10^4^, or 6.0 × 10^3^ conidia/ml suspension. Conidia loads were determined according to the protocol described above, with 6.4 × 10^7^ (high dose) and 6.2 × 10^4^ (low dose) producing the mass-corrected conidia loads (5.1 × 10^4^ ± 1.5 × 10^4^, *n* = 10 termites; 3.2 × 10^4^ ± 1.3 × 10^4^, *n* = 10 termites, respectively).

### Conidia exposure treatments

To determine the effect of fungal exposure on survival, *I. schwarzi* (instars 6, 7 and nymphs) were exposed to a high (6.4 × 10^7^ conidia/ml) or low dose (6.2 × 10^4^ conidia/ml) of *M. anisopliae* conidia according to the abovedescribed procedure. Immediately after exposure, individual termites were transferred haphazardly into sterile Petri dishes (60 × 15 mm) lined with filter paper (Whatman Qualitative no. 1) moistened with 150 µl sterile water (low dose, *n* = 25; high dose, *n* = 25). To examine the effect of group size on survival, subcolonies containing mixed instar groups of 10 (low dose, *n* = 5; high dose, *n* = 5) and groups of 25 (low dose, *n* = 5; high dose, *n* = 5) were similarly established. This experiment used 123 *I. schwarzi* termites each from three colonies (A, B, and C). Colony D, due to its larger size, provided 231 termites. Control termites from all four stock colonies were treated with a conidia-free 0.1% Tween 80 suspension medium and established in Petri dishes containing an isolated termite (*n* = 25) or mixed-instar groups of 10 (*n* = 5) or 25 (*n* = 5). All Petri dishes were subsequently stacked in covered plastic boxes (30 × 23 × 10 cm) and maintained in the laboratory.

### Survival

All termites were censused daily for 20 days following exposure, providing survival data to estimate immune function (Boots and Begon 1993; Moret and Schmid-Hempel 2000; Armitage et al. 2003). Dead individuals were removed, surface sterilized with 5.2% sodium hypochlorite, rinsed twice with sterile water, and plated on potato dextrose agar to confirm that mortality was due to infection by *M. anisopliae* (Rosengaus et al. 1998). Confirmation rates for conidia-exposed termites ranged from 92% to 100% while the confirmation rate for controls was zero.

### Statistical analysis

To determine the effect of conidia exposure on survivorship, several survival parameters were estimated, including the survival distribution (the time-course of survival), percent survivorship, and median survival time (LT50). A Cox Proportional Regression Analysis was performed to determine the relative hazard ratio of death. The model included the following variables: group size (1, 10, or 25 individuals), exposure (high dose, low dose, or control), and species (*I. schwarzi* or *Z. angusticollis*). The resulting relative hazard functions characterized the instantaneous rate of death at a particular time, given that the individual survived up to that point, while controlling for the effect of other variables on survival (SPSS 1990; Rosengaus et al. 1998). Survival distributions were analyzed with the Breslow Statistic (BS; Kaplan-Meier Survival Test, SPSS 1990). When multiple, pairwise comparisons were made, the α-value of significance was adjusted (Rice 1989). Data derived from Rosengaus et al. (1998) was used to compare the survivorship of *I. schwarzi* to that of *Z. angusticollis* following exposure to masscorrected doses of conidia.

## Results

An overall Cox Proportional Regression Analysis showed that conidia dosage, group size and species were all significant and independent predictors of termite survival [Wald Statistic (WS) = 311, 216, and 44, respectively; p < 0.001]. After controlling for the effects of all other variables in the model, isolated termites had 5.5 times the hazard ratio of death relative to grouped termites (WS = 214, df = 1, p < 0.0001), while termites in groups of 10 did not differ significantly from groups of termites composed of 25 individuals (WS = 3.3, df = 1, p = 0.07). Furthermore, *Z. angusticollis* had a significantly higher hazard ratio of death (1.6 times higher) relative to that of *I. schwarzi*, even after controlling for the influence of group size and conidia exposure on survivorship. The effects of group size and species are discussed in detail below.

**Figure I.**
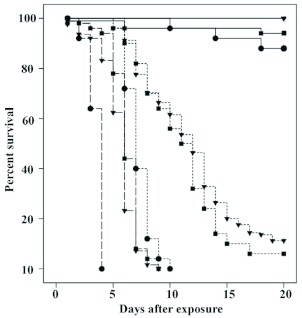
Survival distributions of *lncisitermes schwarzi* maintained in isolation (circles 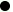), groups of 10 (squares 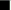), or groups of 25 (inverted triangles 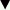) following exposure to a low (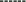) or high (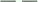) dose of conidia/ml, or control (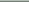). High quality figures are available online.

### Susceptibility of *I. schwarzi* to fungal infection

Survival analyses and the various estimated survival parameters provided further support for the significance of the role that group living in *I. schwarzi* has on the control of fungal disease. *I. schwarzi* exhibited dosage dependent mortality within each group size, but the effect of disease was significantly more pronounced when termites were isolated than when maintained in groups of 10 or 25 (Figure 1 and Table 1). Termites kept in groups of 25 had an 83% reduction in the hazard ratio of death relative to isolated termites. Interestingly, colony of origin and instar (an estimator of age) were not significant predictors of *I. schwarzi* survival (Wald Statistic = 0.2, 0.4; df = 3,1; p > 0.05, respectively).

### Interspecific variation in susceptibility

**Figure 2.**
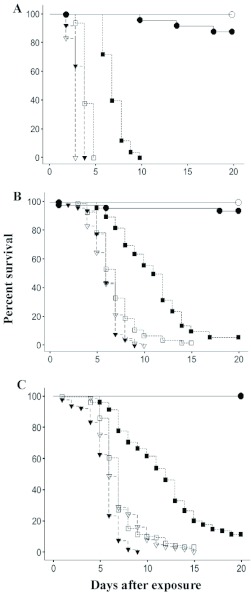
Survival distributions as a function of termites maintained in isolation (A), groups of 10 (B), or 25 individuals (C) of *lncisitermes schwarzi* (closed symbols), and *Zootermopsis angusticollis* (open symbols), following exposure to a low (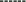) or high (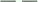) dose of conidia. Control: (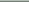).

Following an exposure to a low or high dose of fungal conidia, isolated *I. schwarzi* survived significantly better than isolated *Z. angusticollis* (BS = 53.4, p < 0.001; BS = 7.0, p = 0.008, respectively; *Z. angusticollis* data from Rosengaus et al. 1998) surviving approximately 1 and 4 days longer following low and high dose exposures, respectively (Figure 2A). Control *I. schwarzi* and *Z. angusticollis* had similar survival distributions (BS = 4.3, p = 0.04; Figure 2A). The above significance values reflect p-value adjusted for multiple comparisons of p = 0.008.

**Table I.**
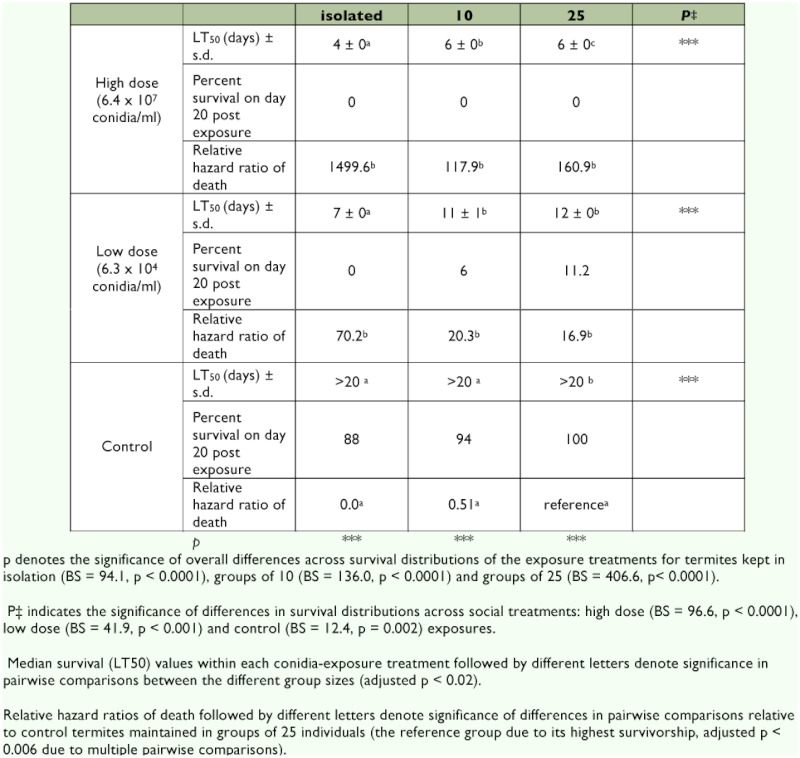
Survival of isolated and grouped *I. schwarzi* according to conidia exposure treatments.

When *I. schwarzi* and *Z. angusticollis* were maintained in groups of 10 individuals, following exposure to the low conidia dosage, *I. schwarzi* survived significantly longer than *Z. angusticollis* (BS = 39.5, p < 0.001; Figure 2B). However, no significant differences were recorded between the two species in either the control treatment or the high conidia dose (Figure 2B). The above significance values reflect p-value adjusted for multiple comparisons at p = 0.008.

Finally, for termites maintained in groups of 25 after exposure to a low conidia dose, *I. schwarzi* also survived significantly longer than *Z. angusticollis* (BS = 98.8, p < 0.001, Figure 2C). But, following a high conidia exposure, *Z. angusticollis* survived significantly longer than *I. schwarzi* (BS = 25.9, p < 0.0001; Figure 2C). The above significance values reflect a p-value adjusted for multiple comparisons of p = 0.008.

### Discussion

Significant interspecific variation in immunocompetence has been described (reviewed in Fellowes and Godfray 2000; Wilson et al. 2000), but immune function has been assessed without challenging hosts with live pathogens or examining survivorship. Schmid-Hempel and Loosli (1998) demonstrated interspecific differences in mortality following exposure to a novel pathogen, but the ecological correlates of immunity remain unknown. There are compelling ecological and evolutionary reasons for predicting that *Z. angusticollis* should be less susceptible to fungal infection than *I. schwarzi*. The nesting and feeding habits of both termite species appear to promote differential growth of microbial communities (Hendee 1933, 1934) and thus differences in encounter rates with disease. The dampwood termite *Z. angusticollis* has significantly higher cuticular and nest microbial loads than the drywood termite *I. schwarzi* (Rosengaus et al. 2003) and, therefore, should be under greater selection pressure to invest more heavily in immune function. Indeed, molecular analyses suggest that antifungal peptides have diversified in response to microbe-related variation in nesting ecology and pathogen pressure in other termite species (Bulmer and Crozier 2004). It is likely that dampwood termites have a longer coevolutionary history with *M. anisopliae* than drywood termites. *M. anisopliae* conidia require high humidity to germinate (Milner et al. 1997), and the moist nest and soil conditions surrounding the decayed wood nests of *Z. angusticollis* are more suitable for the development of this fungus than the dry wood environments of *I. schwarzi.* Thus, it is conceivable that coevolution between *Z. angusticollis* and *M. anisopliae* would have resulted in greater immune adaptation to resist *M. anisopliae* infection rather than for *I. schwarzi*, to which the pathogen may be novel. Yet the fact that the latter species had higher survival across most treatments (with the exception being when termites were maintained in groups of 25 individuals following exposure to the high conidia dosage) does not support the hypothesis that adaptive variation in immune response results from heterogeneity in microbial pressures. Differences in cuticular chemistry may also influence the susceptibility of *I. schwarzi* to *M. anisopliae.* It would be expected that *Z. angusticollis*, with their apparently more heavily melanized cuticle, would be more resistant to fungal infection although other substances distributed on the cuticle could impact microbes. Another plausible explanation for the lack of a consistent association between susceptibility to fungal infection and microbial loads associated with the different nesting and feeding habits of *Z. angusticollis* and *I. schwarzi* is that the methods for estimating microbial loads in termite colonies may not have a level of resolution sufficient to identify interspecific differences in pathogenic and/or parasitic forms (Cruse 1998; Rosengaus et al. 2003). Records of colony forming units isolated from termite and nest washes provide only a one-time snapshot of culturable nest microbes. Ultimately, molecular immunity may be driven by the presence and abundance of pathogenic/parasitic microorganisms that vary temporally throughout colony ontogeny. Unfortunately, comparative quantitative analyses on the abundance of pathogenic/parasitic microorganisms are lacking.

These results illustrated the importance of sociality in coping with disease and parasitism (Rosengaus et al. 1998, 2000; Rosengaus and Traniello 2001; Traniello et al. 2002; Shimizu and Yamaji 2003; Maekawa et al. 2005; Calleri et al. 2006; Wilson-Rich et al. 2007; Yanagawa and Shimizu 2007). An emerging literature shows that termites, independent of species, benefit from group living when exposed to a variety of infectious agents including entomopathogenic fungi and nematodes. Interspecific differences in behaviors such as allogrooming, known to be associated with the social control of disease, may be significant in determining resistance to infection.

Disease has been proposed as an important selective factor in termite evolution (Rosengaus and Traniello 1993; Thorne and Traniello 2003). Selection for individual physiological resistance was perhaps influenced more by group living than by ecological variations in exposure to antigens. Calleri et al. (2006) demonstrated that low genetic heterozygosity reduced the disease resistance of grouped *Z. angusticollis*, but did not appear to negatively affect the immune response of individual termites maintained in isolation. This suggests that social mechanisms of infection resistance may be more significant in termite disease control than individual physiological immunity and its underlying genetic architecture. In other words, socially mediated immunocompetence (Traniello et al. 2002), may have benefits in disease resistance sufficient to relax selection for individual immune function. Research linking ecological heterogeneity in pathogenic pressure, genetic variation in immunity, and direct measurement of *in vivo* immune response to both inert and viable disease agents is required to further evaluate this hypothesis.
